# Journey to the heart of macrophages: the delicate relationship between HIV-1 and a multifaceted cell type

**DOI:** 10.1186/1742-4690-7-28

**Published:** 2010-04-07

**Authors:** Andrea Cimarelli

**Affiliations:** 1 LaboRetro, Department of Human Virology, Ecole Normale Supérieure de Lyon, Lyon, France; 2 INSERM, U758, Lyon, France; 3 University of Lyon 1, IFR128 BioSciences Lyon-Gerland, Lyon-Biopole, Lyon, France

## Abstract

Cells of the monocyte-macrophage lineage play multiple roles during the infection of primate lentiviruses serving as reservoirs for viral production or as vectors for viral spread to other cells and tissues. The human immunodeficiency type I virus is not only capable of establishing such complex and dynamic relations with this cell type, but is also able to modulate their physiology and behavior, thus shaping ensuing cellular immune responses. In this issue of Retrovirology, a series of reviews explores the multiple manners in which the virus and cells belonging to the monocyte-macrophage lineage interact and affect each other.

## Introduction

This issue of Retrovirology presents a series of reviews centered on the complex relationship established between monocytes/macrophages and the human immunodeficiency type I virus (HIV-1). This cell type plays multiple and important roles during viral replication and pathogenesis serving as a haven for the multiplication of the virus, as a vehicle for its spread into privileged sites, as a cell type to take over and modify host immune responses. The reviews presented here deal extensively with all these issues, leading the reader to appreciate the prominent role of macrophages during HIV-1 induced pathogenesis.

## Discussion

Macrophages are resident cells that differentiate in tissues upon migration of circulating blood monocytes. Migration can occur through the blood brain barrier accounting for the passage of HIV-1 into the central nervous system or to sites of infection. Tissue residency is accompanied with the differentiation of monocytes in macrophages, a differentiation that is heavily dependent on the environment in which the cells find themselves. There, as professional antigen presenting cells (APCs), macrophages establish numerous contacts with T cells and participate with cytokine secretion to mount appropriate immune responses.

It seems clear that monocytes and by extension macrophages represent a heterogeneous cell population that includes cells of different functionalities. At least two major monocyte populations exist that are characterized by the surface expression of the CD14 and CD16 markers (either CD14^+^CD16^- ^or CD14^+^CD16^+^), and evidence for a differential behavior of these two cell populations with respect to HIV-1 clearly exist [1]. To add to their heterogeneity, differentiation of monocytes into macrophages can be accomplished following a number of stimuli. Monocytes do not simply differentiate into a single type of macrophage, but do so via concomitant polarization, that is through the specific differentiation into macrophages of specific functionalities. Schematically, this polarization can lead to macrophages with pro- or anti-inflammatory and tissue repair properties defined as M1 or M2 macrophages by analogy with the Th1 and Th2 nomenclature of helper T cells [2], but it is likely that this represents a simplification of a more plastic polarization system.

In this cell type, or rather in these cells which are so similar yet so different, HIV-1 replicates. HIV-1 is not alone among lentiviruses to infect monocytes/macrophages. The Visna/CAEV and the equine infectious anemia (EIAV) viruses display an exquisite, even more restricted, preference for this cell type [3,4], possibly underlying the important role that myeloid cells have in lentivirus infection.

Despite the fact that circulating monocytes are rather resistant to HIV-1 infection, these cells bear HIV-1 *in vivo *and piggyback the virus into tissues and through the blood brain barrier into the central nervous system [5,6]. With their differentiation into macrophages, and according to the stimulation received, macrophages become permissive to HIV-1, and albeit remaining more restrictive to the virus than other cell types, become overtly infected. The virus takes hold of these cells and uses them to spread to neighboring T cells, to support a low persistent level of virus production, as well as to influence the cytokines secreted by these cells.

If some of these properties can be shared with T cells, macrophages display peculiar properties with which the virus is confronted, and the reviews presented here clearly depict these differences. Two reviews, from Ayinde and from Benaroch and colleagues explore the specificities of macrophages with respect to different aspects of the viral life cycle [7,8]. The first details the latest findings on the role that the non-structural viral proteins Vpr and Vpx (the first conserved among all primate lentiviruses; the second coded almost exclusively by members of the HIV-2/SIV_SM _lineage) play during the early phases of infection of macrophages, while the second provides a thorough description of the process of virion particle production (i.e. virion assembly). A third review by Bergamaschi and Pancino more globally outlines the overall blocks that hinder the life of HIV-1 inside monocytes and macrophages [9].

The molecular mechanisms with which a viral reservoir is established in macrophages is reviewed by Le Douce and colleagues [10], while the mechanism with which monocytes allow entry of HIV-1 into the central nervous system, where the virus causes a series of neurological disorders collectively named HIV encephalitis (HIVE) is described by the accompanying review of Gras and Kaul [11]. Finally, the interplay between macrophage polarization and the effect that different viral proteins exert on the activation status of macrophages are described in two reviews by Herbein and Varin, and Herbein and colleagues [12,13].

## Conclusions

The reviews presented in this issue of Retrovirology explore a number of interesting issues and collectively concur in depicting a comprehensive overview of the delicate relationship established between macrophages and HIV-1.

## Competing interests

The author declares that they have no competing interests.
